# Permeability of ergot alkaloids across the blood-brain barrier in vitro and influence on the barrier integrity

**DOI:** 10.1002/mnfr.201100431

**Published:** 2011-12-07

**Authors:** Dennis Mulac, Sabine Hüwel, Hans-Joachim Galla, Hans-Ulrich Humpf

**Affiliations:** 1 NRW Graduate School of Chemistry University of Münster, Münster, Germany; 2 Institute of Food Chemistry, University of Münster, Münster, Germany; 3 Institute of Biochemistry, University of Münster, Münster, Germany; Westfälische Wilhelms-Universität Münster, Institute of Food Chemistry, Corrensstrasse 45, 48149 Münster, Germany, **Fax:** +49-251-8333396

**Keywords:** Active transport, Blood-brain barrier, Ergot alkaloids, Permeability, TEER

## Abstract

**Scope:**

Ergot alkaloids are secondary metabolites of *Claviceps* spp. and they have been in the focus of research for many years. Experiments focusing on ergotamine as a former migraine drug referring to the ability to reach the brain revealed controversial results. The question to which extent ergot alkaloids are able to cross the blood-brain barrier is still not answered.

**Methods and results:**

In order to answer this question we have studied the ability of ergot alkaloids to penetrate the blood-brain barrier in a well established in vitro model system using primary porcine brain endothelial cells. It could clearly be demonstrated that ergot alkaloids are able to cross the blood-brain barrier in high quantities in only a few hours. We could further identify an active transport for ergometrine as a substrate for the BCRP/ABCG2 transporter. Investigations concerning barrier integrity properties have identified ergocristinine as a potent substance to accumulate in these cells ultimately leading to a weakened barrier function.

**Conclusion:**

For the first time we could show that the so far as biologically inactive described 8-(*S*) isomers of ergot alkaloids seem to have an influence on barrier integrity underlining the necessity for a risk assessment of ergot alkaloids in food and feed.

## 1 Introduction

Ergot alkaloids are a variety of secondary metabolites of the fungal family *Claviceps*, with the most notable species in Europe *Claviceps purpurea* [1–3]. With the infection of different grain species, especially rye, the toxic alkaloids are accumulated in the reservoir of the fungus, the sclerotia, and can contaminate different cereal products. With more than 40 toxic alkaloids, belonging to different groups, a wide range of toxic effects were reported after consumption of contaminated food and feed [2]. These intoxications are known since early history of man even referring to a time BC. Different toxic effects are mentioned so far with the first documented case of ergotism (St. Anthonys fire) in mediaeval times [4]. Acute toxic symptoms range from a rise in blood pressure, to vasoconstriction, with gangrene of extremities and ultimately the loss of related body parts. Furthermore, neurotoxic symptoms are described with spasms, hallucinations, delirium or epileptical fits [5,6].

**Figure 1:**
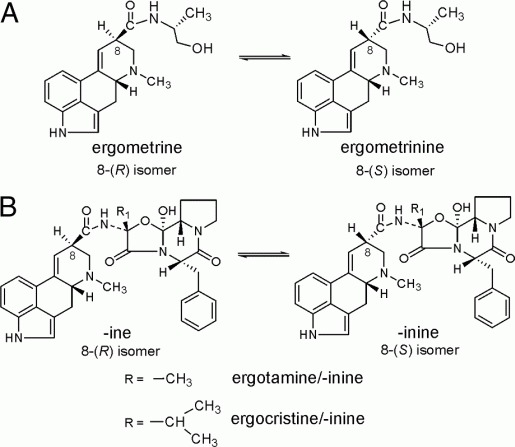
Structure and formation of equilibrium of (A) ergometrine/ergometrinine and (B) peptide ergot alkaloids: ergotamine and ergocristine (with corresponding/-inine forms).

Out of the variety of substances in the sclerotia six have been identified as mainly dominant and relevant for toxic effects. Therefore, the alkaloids ergometrine (lysergic acid amide alkaloid), ergocristine, ergotamine, ergocornine, α-ergocryptine and ergosine (peptide ergot alkaloids) are of primary interest [7]. All of these substances contain an optically active carbon atom at position C-8 and therefore show an epimerization effect under light, pH value change, higher temperature or in aqueous solvents [8,9]. Consequently, the occurrence of each single compound out of these six is referred to two different isomers, called lysergic 8-(*R*) and isolysergic 8-(*S*) isomer, with chosen suffixes -ine and -inine, shown in Fig. 1 [9,10]. It is reported that 8-(*S*) forms of ergot alkaloids are less biologically active [11].

Nevertheless, for a risk assessment these six compounds have to be considered along with their corresponding -inine form, because each isomeric form can be converted into each other. Currently there is no legal regulation for the ergot alkaloid content in food and feed only for ergot impurities in feed materials [12].

Different animal experiments lead to several LD_50_ values for the different ergot alkaloids in a range of 0.9–275 mg/kg b.w. depending on used species and application methods [3]. Besides this aspect, an interaction with several neurotransmitters is reported. Due to their structural similarity with substances such as dopamine, serotonin or norepinephrine they show agonistic and antagonistic abilities with these receptors, ultimately causing the described toxic symptoms in humans [13,14].

Toxic properties are not the only ones described in the literature for ergot alkaloids. Early formulations of sclerotia were used as a drug for migraine therapy. Later the main component ergotamine was isolated from the mixture (1918) and was used for a long time in migraine therapy since 1926 [15]. Although a clear interaction of ergotamine with neurotransmitters is reported, the responsible mechanism is still controversial [16,17]. Peptide ergot alkaloids like ergotamine show a low oral bioavailability, less than 2% in humans [18]. Therefore, different studies were performed using various application forms of ergotamine and have produced controversial data. The study of Ala- Hurula et al. with ten patients has shown the ability of ergotamine to reach the cerebrospinal fluid (CSF) in a concentration of 0.4 ng/mL. Plasma levels of ergotamine after an oral dose of 2mg were measured in the same range [19]. A similar study of Hovdal et al. with 18 patients and different application methods has shown no ergotamine in CSF [17]. Possible explanation in the literature refers to methodological considerations, due to the detection limit of the assay [20]. Consequently, the question whether ergot alkaloids are able to reach the brain remains unclear, although it is an empirical fact, known from hallucinations, spasms and the treatment of Parkinson patients. As alkaloids act as the pharmaceutical (and toxic) compounds, the xenobiotic substances have to cross the blood–brain barrier to have an impact on the central nervous system (CNS). This special arrangement of highly specialized cells, namely brain capillary endothelial cells, regulates the transfer between substances from the blood flow and the brain interior [21]. This cell monolayer restricts other compounds by a very specialized cell–cell contact (tight junction) to exclude any paracellular diffusion for hydrophilic compounds [22]. To restrict hydrophobic compounds special efflux/influx transport proteins regulate the access to the brain compartment [23]. To mimic the blood–brain barrier, a validated model was used which is based on porcine brain capillary endothelial cells (PBCECs) seeded on a Transwells filter system [24]. With the addition of hydrocortisone at physiological concentrations, barrier integrity, represented by transendothelial electrical resistance (TEER) of more than 1000Ω*cm^2^could be measured [25]. With the use of this model system, a very good comparison to the in vivosetting (1800Ω*cm^2^) is possible [26].

To answer the question whether ergot alkaloids like ergotamine are able to cross the blood–brain barrier in vitro, the described model system was used. To assay the effect of the ergot alkaloids on barrier integrity the parameters, cytotoxicity, TEER, as well as ^14^C-sucrose permeability were measured during or after incubation with alkaloids. In order to study the ability to cross the endothelial barrier, the permeability of three model compounds, ergotamine, ergocristine and ergometrine, was determined. In addition to this, transport studies were performed to evaluate any active transport properties, including inhibitor experiments.

## 2 Material and methods

### 2.1 Chemicals

All used ergot alkaloid standard substances were purchased from Alfarma (Cernosice, Czech Republic) with the exception of ergotamine-D-tartrate and ergometrine-maleate, which were ordered from Sigma-Aldrich (Steinheim, Germany). Methysergid-maleate was ordered from Biotrend (Wangen, Switzerland). All chemicals, cell culture medium and supplements were obtained from Merck (Darmstadt, Germany), Sigma-Aldrich or Biochrom (Berlin, Germany).

### 2.2 Primary cultures of PBCECs

Isolation of primary PBCECs was performed according to the established model system, described by Franke et al. [24].

Cryopreserved cells were prepared according to the described methods [27–29]. Briefly, cells were seeded (250 000 cells/well) on rat tail collagen (0.54 mg/mL)-coated microporous 12-well Transwell® filter system (1.12 cm^2^growth area, day in vitro 2 since preparation (DIV 2)) in plating medium (Medium M199 with Earl's salts, 0.7mM glutamine, 100 mg/mL penicillin/streptomycin, 100 mg/mL gentamycin, 10% (v/v) newborn calf serum (NCS)) on the apical (upper) compartment, reflecting the blood compartment in contrast to the basolateral compartment, reflecting the brain side. Cells were maintained for two days and to induce in vivo phenotype, the medium was changed to serum-free medium (DIV 4, Dulbecco's modified Eagle's medium/Ham's F-12, 4mM glutamine, 100 mg/mL penicillin/ streptomycin, 100 mg/mL gentamycin) supplemented with 550nM hydrocortisone corresponding to the physiological concentration [25]. All experiments were performed after another two days of differentiation phase (DIV 6).

Ergot alkaloids were applied in serum-free medium from a stock solution (2mM in ethanol/0.25 g/L tartaric acid 40:60 (v/v)). Correction factors were used to guarantee equilibrium of -ine and -inine forms as described in an earlier report [30]. All ergot alkaloids were incubated as a mixture of both isomeric forms, if not mentioned otherwise.

### 2.3 Barrier integrity studies

#### 2.3.1 Determination of cytotoxicity (CCK-8 assay)

For evaluation of general cytotoxic effects, the Cell Counting Kit-8 (CCK-8) from Dojindo Laboratories was used (Tokyo, Japan). Cells were seeded on Collagen-G-coated 96-well plates (250 000 cell/cm^2^) and were treated the same way like on Transwell® filter system (DIV 2 plating medium and DIV 4 serum-free medium for 2 days). Cells were incubated with various concentrations of ergot alkaloids (0.001–20 mM) for 24 h. After toxin exposure, the WST-8 (2-(2-methoxy- 4-nitrophenyl)-3-(4-nitrophenyl)-5-(2,4-disulfophenyl)-2*H*tetrazolium, monosodium salt) solution was added. The generated amount of formazan dye (reduction in tetrazolium by active dehydrogenases in viable cells) is directly proportional to the amount of viable cells. The absorbance of each well was measured using an FLUOstar Optima microplate reader (BMG Laboratories, Jena, Germany) at 450 nm. The absorbances of the ergot alkaloid treated wells were compared with a solvent control.

#### 2.3.2 TEER measurement

TEER was used as a parameter for barrier integrity [31], measured by the cellZscope® device (nanoAnalytics, M. unster, Germany). The module used was suitable for 24 Transwells filter system, grown with PBCEC monolayer. For all experiments only cell monolayers with a TEER value above 600Ω*cm^2^were used. Overall, the used cell monolayers had shown TEER values between 600 and 2000Ω*cm^2^. TEER was checked and recorded for all permeability or transport measurements (2.4.1 and 2.4.2).

#### 2.3.3 14C-sucrose permeability

To verify the barrier integrity results of the TEER analysis, experiments with radiolabeled ^14^C-sucrose were performed. Due to the fact that no transporter system is present for sucrose and no uptake could be evaluated, the permeability of sucrose is a suitable model substance for the barrier integrity [32]. Therefore, 1–10 μM of used ergot alkaloids were applied in apical and in basolateral compartment (a/b) for 24 h. After incubation radiolabeled ^14^C-sucrose was added to the apical side to evaluate the effect of the ergot alkaloids in correspondence with the TEER value. The exact procedure was performed according to [33]. In a timedependant experiment, the permeability was determined as described in Section 2.4.1.

#### 2.3.4 Fluorescence microscopy

Cells were seeded and cultivated as described before. After 24 h of incubation with ergot alkaloids, the medium was removed, cells were fixed using 4% paraformaldehyde (v/v) and the primary antibody was applied (1 mg/mL anti-occludin, Zytomed, Berlin, Germany in 0.5% BSA (w/v)) for 30 min at 371C. The fluorescence-labeled secondary antibody (2 mg/mL Alexa Fluors 546 goat anti-mouse (Invitrogen, Paisley, UK), in 0.5% bovine serum albumin in PBS (w/v)) was added like before. Visualization of cell nuclei was performed with a brief incubation of Hoechst 33258 (Bisbenzimide, 1 mg/mL, Sigma-Aldrich). After intensive washing with PBS, filters were cut out and mounted in Aqua Poly/Mount (Polysciences, Washington, USA). The microscopy was perofrmed after a drying period of 24 h.

### 2.4 Permeability and transport measurements

#### 2.4.1 Permeability

Permeability studies were carried out using 3–4 cell monolayers in at least three individual preparations with three control filters (containing equal amount of solvent). Ergot alkaloid solutions were added to either the apical (a) or basolateral (b) compartment in tenfold higher concentrations by exchanging part of the serum-free media (10–100 mM). After several time points (1, 2, 3, 6, 12 and 24 h) samples were taken from both sides with 10–20 mL depending on the used concentration. The collected samples were diluted with an internal standard methysergid-maleate (final concentration: 162 ng/mL) and were analyzed by high-performance liquid chromatography (HPLC, see Section 2.6). Uptake of substances was determined after lysis of filters in 1% Triton X-100 solution. To guarantee barrier integrity, TEER values were recorded simultaneously.

Permeability coefficients were calculated using the equation


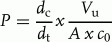


with *d*_c_/*d*_t_ as the change of concentration in acceptor compartment, *V*u the volume of acceptor compartment, *A* the membrane surface and *_c_*0 the initial concentration used [34].

To exclude the effect of the polycarbonate membrane on the permeability of ergot alkaloids, the *p*-values were corrected by performing experiments without cells. The results obtained were corrected using


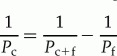


with *P*c as the value of the cell monolayer, *P*f representing the *p*-value of the polycarbonate membrane and *P*_c+f_ as the *p*-value from cell monolayer and membrane [34].

#### 2.4.2 Active transport

Active transport studies were performed by the addition of equal concentrations of ergot alkaloids in both compartments (a/b; 10–100 mM, final concentration: 1–10 mM). Samples were collected after the same time points as presented in Section 2.4.1 with an additional sample taken at 48 h. The quantification was performed as described in Section 2.6.

To verify an active transport, PSC 833 (inhibitor P-glycoprotein [35]) and Fumitremorgin C (inhibitor BCRP/ ABCG2 [36]) were preincubated for 1 h in both compartments at a final concentration of 10 μM each.

### 2.5 Quantification of Ergot alkaloids

For quantification analysis of ergot alkaloids, an HPLC system coupled with fluorescence detection was used. Separation of the substances was performed using a 250mm × 4.6mm id, 4.6 mm, Varian OmniSphers C18 column (Darmstadt, Germany) with a binary pump (Merck- Hitachi L-7100, Tokyo, Japan) and sample injection (20 mL) with a Merck Hitachi autosampler AS2000A. The substances were detected with a Merck-Hitachi FLD F-1050 fluorescence detector (excitation: 330 nm, emission: 415 nm). Mobile phase consisted of acetonitrile (solvent A) and ammonium carbamate buffer 0.2 g/L (solvent B) at a flow rate of 1 mL/min.

Ergotamine/-inine and ergocristine/-inine were separated using a gradient starting at 60% A for 5.5 min increasing to 80% A in 2.5 min, holding these conditions for 2 min. The system was equilibrated for 4 min with a total run time of 14 min. Ergometrine/–inine was separated using a step gradient starting at 27% A for 2.5 min, changing to 32% A for 2 min and equilibrating the system again (for 8.5 min) with a total run time of 13 min. Data acquisition was performed using the Merck-Hitachi D-7000 HSM HPLC System Manager. Quantification was performed via comparing the peak area of the different alkaloids as an internal standard (final concentration in each sample: methysergid maleate, 162 ng/mL), using different calibration curves (40–400 ng/mL). All quantification results mentioned in this work are sum parameters for the –ine and –inine forms, if not mentioned otherwise.

### 2.6 Statistical analysis

All presented data are given as mean7SEM. For transport and permeability studies, three different cell monolayers were used in at least three different preparations (*n*59). CCK-8 data were measured with a minimum of six wells in three different preparations (*n*518). Differences were determined using the unpaired Student's *t*-test with *p*≤0.05 considered significant.

## 3 Results

### 3.1 Barrier integrity

#### 3.1.1 Cytotoxicity

**Figure 2:**
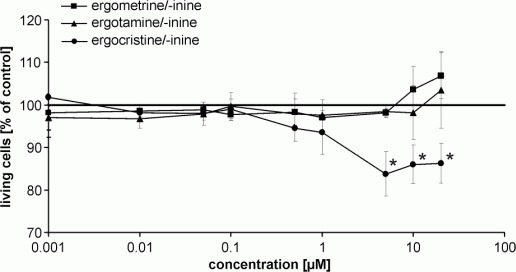
Cell viability of PBCEC determined with the CCK-8 assay after 24 h incubation with ergot alkaloids in concentrations ranging from 0.001 to 20 mM (*n* = 18). Significant results (*p*≥0.05, indicated by *) were obtained for ergocristine/-inine with increasing concentration starting at 5 mM.

Figure 2 shows the viability data for PBCEC after treatment with different ergot alkaloids. For the period of 24 h, small cytotoxic effects of ergocristine/ergocristinine were detectable. Cell viability declines to about 85% of the control with significant differences starting at 5 μM for these compounds.

The other compounds appear to have no impact on cell viability. No EC_50_ values were calculated due to the very low effect of the compounds. For the following permeability and transport studies, the three model compounds were incubated as a sum of 8-(*R*) and 8-(*S*) isomers (Section 2.2): Ergometrine/-inine (EM) as a lysergic acid amide and ergotamine/-inine (ET) and ergocristine/-inine (EC) as the peptide ergot alkaloids.

#### 3.1.2 TEER measurement

**Figure 3:**
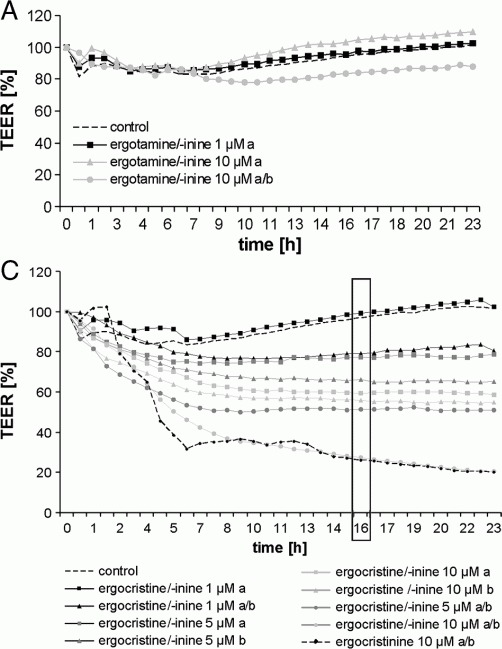
Effect of ergot alkaloids on PBCEC formed barrier, expressed through influence on TEER value during 24h incubation of substances in different compartments (a5apical, b = basolateral, a/b = apical and basolateral). (A) Ergotamine/-inine (ET), (B) ergometrine/- inine (EM) and (C) ergocristine/-inine (EC) in different concentrations ranging from 1 to 10 mM and for (C) additionally dashed line for ergocristinine with 10 mM a/b and (D) TEER value after 16 h incubation with EC and ergocristinine in different concentrations; all: *n*59 with standard error: 10–15%, not shown for A–C.

To guarantee barrier integrity of cell monolayers (≥600Ω*cm^2^) during permeability and transport studies, TEER values were measured during all experiments. Figure 3 shows different application forms (apical5a, or basolateral5b) of the three model compounds. For EM and ET analogue results were obtained as in cytotoxicity experiments (Section 3.1.1). No influence on the barrier integrity was detectable using concentration of 1 μM (a) and 10 μM (a) or equal concentration of 10 μM applied on both sides (a/b) in comparison to the control (Fig. 3A and B). The data were in agreement with measured ^14^C-sucrose permeability as no influence was detectable after incubation with EM or ET (control: 3.6770.48 × 10^−7^cm/s; ET (10 μM (a/b)): 3.92 ± 0.67 × 10^–7^cm/s; EM (10 μM (a/b)): 4.4170.91 × 10^−7^cm/s).

The results obtained for EC showed an influence on barrier integrity using increasing concentrations (Fig. 3C). Using concentrations of 1 μM (a) no effect was detectable, while incubation of 1 μM (a/b) or 5 μM EC (a) decreased barrier integrity to about 80% of initial TEER. A much higher influence was measured, using concentrations of 5 μM (b), 10 μM (a), 10 μM (b) and 5 μM (a/b) with a TEER value of 50–70% of the starting value. The highest effect was measured with concentrations of 10 μM (a/b) of EC, which leads to a time depending disruption of the barrier integrity in the first 6 h resulting in TEER values of only 25% after 24 h (≥300Ω*cm^2^). A plateau of TEER values is reached after about 16 h. To give a better comparison, the TEER values for this time point are summarized in Fig. 3D. Determination of ^14^C-sucrose permeability proved the disruption with a value of 1.5270.45 × 10^–6^cm/s (control: 3.6770.48 × 10^−7^cm/s).

In addition to the measurement of EC, the pure 8-(*S*) isomer, ergocristinine was also incubated with PBCEC monolayer. In contrast to the other used alkaloids, 490% of the initial concentration stays stable in the cell culture medium with no rearrangement of the equilibrium between the two isomers. During incubation with the 8-(*S*) isomer ergocristinine a similar picture occurs, as with the EC mixture. The influence on TEER values was nearly the same and 10 μM ergocristinine (a/b) also leads to a disruption of the barrier, which is representatively shown in Fig. 3C and D.

#### 3.1.3 Fluorescence microscopy

**Figure 4:**
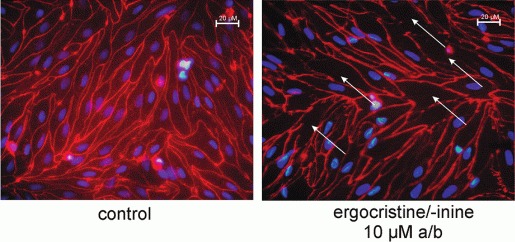
Immunostaining of occludin and cell nuclei in PBCEC after 24 h incubation with 10 mM ergocristine/-inin (EC) apical and basolateral (a/b). Stained occludin with red fluorescence and stained cell nuclei in blue. TEER of control cells >1000Ωcm^2^ in contrast to EC incubated samples <200Ωcm^2^. Regions without cell nuclei are marked with arrows in the EC sample.

Fluorescence microscopy of visualized tight junction protein occludin was used as a third parameter for the influence of ergot alkaloids on PBCEC. As shown in Fig. 4, EC incubated samples (10 μM a/b) differ from control samples. The staining of occludin as a typical tight junction protein shows only a small influence on single areas of the cell monolayer. With the additional cell nuclei staining clear, disrupted cells could be visualized by the absence of nuclei. Similar results were obtained after staining ergocristinine (10 μM a/b) samples (data not shown).

### 3.2 Permeability

Depending on the data from barrier integrity, all permeability experiments were performed using 1 μM of EC and ergocristinine and 10 μM of EM and ET.

**Figure 5:**
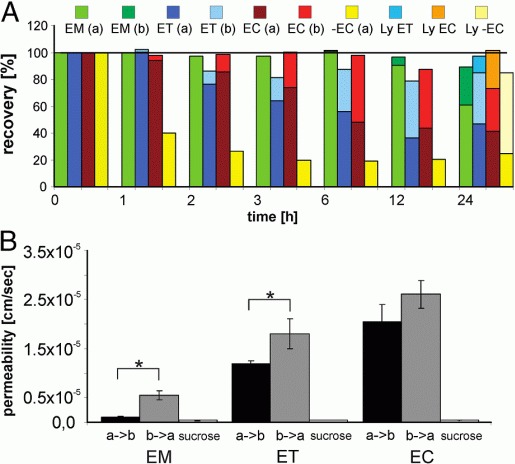
Distribution of ergometrine/-inine (EM, 10 mM), ergotamine/- inine (ET, 10 mM), ergocristine/-inine (EC, 1 mM) and ergocristinine (−EC, 1 mM) after application of alkaloids in the apical compartment of PBCEC monolayers (A). Samples were analyzed after 1, 2, 3, 6, 12 and 24 h for apical (a) and basolateral (b) compartment, as well as cell lysate (Ly) after 24 h with *t* = 0h as 100% value. Calculated permeability coefficients (B) for EM, ET and EC permeation from apical to basolateral compartment (a → b), vice versa (b → a) and for 14C-sucrose permeability after incubation with 10 mM EM (a/b), 10 mM ET (a/b) and 1 mM EC (a/b) as marker for an intact barrier after ergot alkaloid incubation. * indicates significant difference (*p*≥0.05, *n =* 59).

Figure 5A shows the time-dependant distribution of the alkaloids in apical and basolateral compartments after an apical addition. Additionally, Fig. 5B shows the calculated permeability coefficients for an application on either apical or basolateral compartment. Depending on the alkaloid used, different permeation properties were determined. Transfer of EM reached a maximum of 29% after 24 h, but first traces were not detectable before 6 h, whereas ET shows a maximum of permeation after 12 h with 40%. EC concentration reaches its maximum after 6 h in basolateral compartment with 49% and the concentration declines to 32% after 24 h. Missing substances could be detected in cell lysate for ET (average 12%) and EC (average 28%), which is also displayed as a value after 24 h in Fig. 5A. It has to be highlighted, that EC in cell lysate consists predominantly of -inine form.

Comparing these data with the incubation of ergocristinine, no permeation to the basolateral compartment was detectable over a period of 24 h. Nevertheless, added ergocristinine disappeared from apical side, leaving 40% of the initial concentration after 1 h and about 20% from 3 to 24 h. The missing concentration of the alkaloid could be detected in the cell lysate after 24 h with over 60% and no rearrangement of equilibrium, consequently only consisting of ergocristinine.

Permeation experiments were also performed after an addition of the different substances on basolateral side (data not shown). The permeability coefficients (*P*_c_) obtained in both studies are summarized in Fig. 5B and Table 1. For ergocristinine no *P*_c_ values were calculated since the substance does not permeate, neither from apical to basolateral compartment, nor from basolateral to apical compartment, although a rapid uptake in the cells was measured.

**Table 1:** Permeability coefficients *P*c for ergometrin/-inine (EM), ergotamine/-inine (ET), ergocristine/-inine (EC) and ergocristinine permeation from apical to basolateral side (a→b) and vice versa (b→a); all: *n* = 9

	*P*c (cm/s) a→b	*P*c (cm/s) b→a
EM	1.05 ± 0.17×10^−6^	6.05 ± 0.91×10^−6^
ET	1.19 ± 0.06×10^−5^	1.80 ± 0.57×10^−5^
EC	2.05 ± 0.34×10^−5^	2.61 ± 0.28×10^−5^
Ergocristinine	No permeation	No permeation

### 3.3 Active transport study

#### 3.3.1 Transport

**Figure 6:**
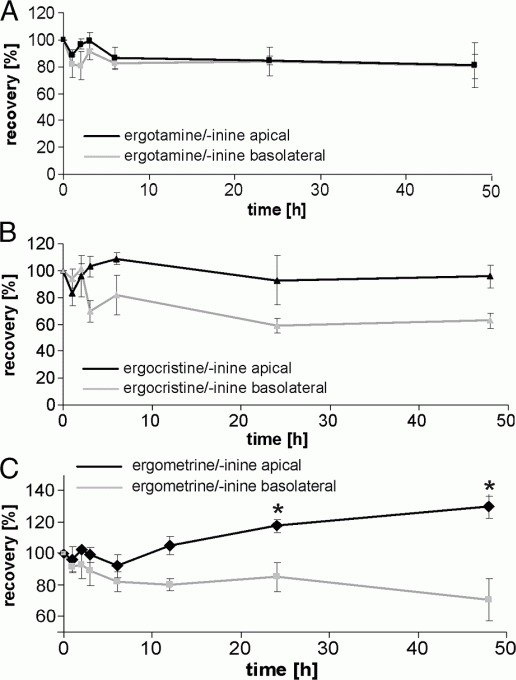
Recovery of (A) ergotamine/-inine (ET, 10 mM), (B) ergocristine/-inine (EC, 1 mM) and (C) ergometrine/-inine (EM, 10 mM) after application of equimolar concentration in both apical and basolateral compartments of the Transwell® system, cultured with PBCEC monolayers. Time-dependant change in recovery in both compartments with concentration at *t* = 0h as 100% value. *indicating significant difference (*p*≥0.05, *n* = 9).

An initial enrichment of substrate in one compartment after the addition on both sides (a/b) leads to the evidence of an active transport of a substance. Figure 6 shows the substrate concentration of EM, ET and EC in apical and basolateral compartments over 48 h. The applied concentrations were 1 μM for EC and 10 μM for ET and EM. For ET and EC no enrichment of substance concentration in one compartment could be detected in this experiment (Fig. 6A and B). The concentration was near the initial concentration (set to 100%) for ET and missing substance could be detected in the cell lysate. Analogue data were obtained for EC, with exception of a decrease in basolateral compartment (60%); however, a high accumulation rate in cell lysate was detectable. Overall, no significant enrichment in one compartment could be observed.

Using EM as a substrate, enrichment on apical compartment was detectable, with a corresponding decrease in basolateral compartment (Fig. 6C). After 12 h the first indications were observed, but not in a significant matter, whereas after 24 h a significant increase to about 117% of the initial concentration was found in the apical compartment (analogue decrease in basolateral compartment). After 48 h a total enrichment of 130% was calculated. In contrast to the other two substrates, no EM was detectable in cell lysate during the whole experiment.

#### 3.3.2 Inhibitor influence

**Figure 7:**
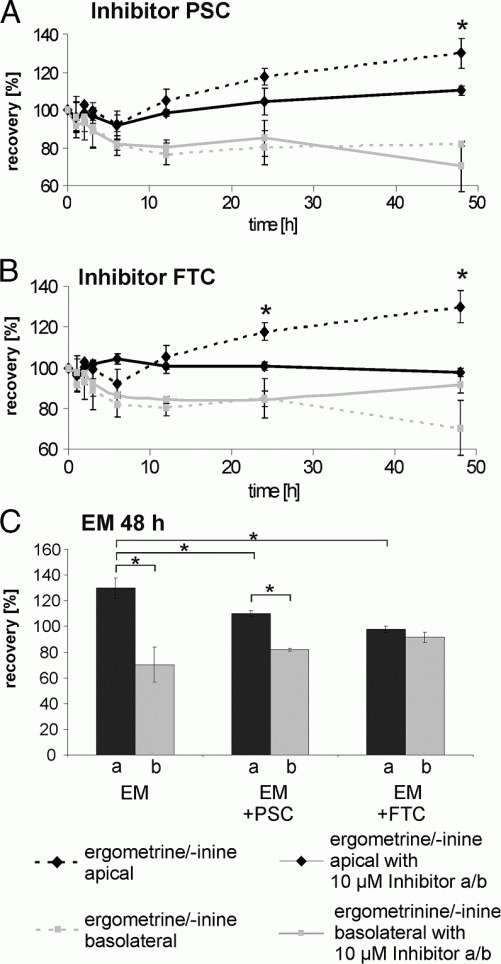
Recovery of ergometrine/-inine (EM, 10 mM) after application of equimolar concentration in both apical (a) and basolateral (b) compartments of the Transwell®* system, cultured with PBCEC monolayers. Influence of co-incubation with (A) PSC (10 mM equimolar apical and basolateral) and (B) FTC (10 mM equimolar apical and basolateral). Dashed line in (A) and (B) reflecting the recovery rate without addition of inhibitor substance and solid line reflecting the recovery rate after addition of inhibitor substance. Time-dependant change in recovery in both compartments with concentration at *t* = 0h as 100% value. (C) As a summary of recovery-rates after incubation of EM (10 mMequimolar a/b) with and without inhibitor substance after 48 h. *indicating significant difference (*p*≥0.05, *n* = 9).

Verification of active transport properties was performed using two different inhibitors: PSC 833 (PSC) and Fumitremorgin C (FTC). Figure 7 shows the influence of the two different inhibitors on the transport of EM. With the addition of PSC a lagging of enrichment in the apical compartment was detected (Fig. 7A). The curve resembles the previous one (3.3.1) but increases only to 110% of the initial concentration after 48 h. Significant differences were only observed at the last measured time point 48 h.

The addition of FTC to EM leads to a total inhibition of the transport process (Fig. 7B). The recovery rates were always determined with about 100% of the initial concentration on the apical side and were significant different in comparison with the previous obtained results (Section 3.3.1). The results for 48 h are summarized in Fig. 7C for EM and the inhibitor influences.

## 4 Discussion

### 4.1 Barrier integrity

The primary toxic effects of ergot alkaloids are referred to an interaction with different neurotransmitters, especially in the brain [13]. In addition, toxic effects like apoptosis are described for ergot alkaloids in human astrocytes [30]. Therefore, we investigated the ability of ergot alkaloids to pass the blood–brain barrier, as well as to influence the barrier properties. Time-dependant experiments were performed whereby TEER values of PBCEC monolayers were the primary parameter to determine any barrier influencing effects during all experiments.

With regard to the CCK-8 assay, the compound ergocristine (in equilibrium with the corresponding -inine form) has shown slight cytotoxic properties, with a significant reduction in cell viability at 5 μM to 85%. The other tested compounds ergotamine and ergometrine (all with corresponding -inine form) had shown no impact in the test system up to a concentration of 20 mM. Out of the six compounds recommended by the EFSA, ergometrine/-inine was chosen for further experiments, because of its structural difference as a lysergic acid amide in contrast to the other substances. Ergotamine/-inine and ergocristine/-inine were used as model compounds, since they differ only slightly in their toxicity, but are nearly structurally identical. It is also reported, that the main alkaloid composition of sclerotia is up to 58% ergotamine and ergocristine, along with their corresponding 8-(*S*) isomers [37,38].

The results from TEER measurements with EM and ET appeared to have no impact on barrier integrity in concentrations up to 10 μM (a/b), independant from incubation side. These results were confirmed by no change in the ^14^C-sucrose permeability after incubation with ergot alkaloids EM and ET. Effects could only be measured using EC (mixture of isomers) or pure ergocristinine (8-(*S*) isomer). In a concentration and compartment dependant matter, the barrier integrity was disturbed. After about six hours, the impact on barrier integrity was simultaneously disrupted for all used conditions, especially using 10 μM (a/b) resulted in a decrease of the TEER values to about 25% (Fig. 3D), leaving a weakened barrier with 100–300Ω*cm^2^(depending on starting value). This effect is also reflected by ^14^C-sucrose permeability (see Section 3.1.2). The impact on the barrier integrity seems to be more efficient with an incubation in the basolateral compartment, or respectively on both sides. The same influence was measured using pure ergocristinine as a substrate. Nearly, the same curves were obtained (only data for 10 μM a/b shown in Fig. 3, Section 3.1.2). Neither data concerning the stability of ergocristinine in cell culture medium, nor concerning the biological activity was previously reported in the literature. In the literature, 8-(*S*) isomeric forms are always mentioned as weakly active compounds [11] only effective due to a rearrangement of equilibrium of the substance [9]. Our data clearly show that ergocristinine is a nearly stable compound (490%), with an effect identical EC.

The impact on barrier integrity was also visualized for EC (10 μM a/b) (Section 3.1.3) after staining of tight junction protein and cell nuclei. Tight junction proteins like occludin are a very good marker for barrier integrity, because of their role as a functional unit [22]. Large sections remain intact, while some small parts of the cell monolayer were destroyed by EC, resulting in areas without cell nuclei, and disruption of occludin-staining, as presented in Fig. 4. These results are in agreement with cytotoxicity data obtained in CCK-8 assay. These holes were found for EC, as well as for ergocristinine incubated samples, indicating the same effect.

The results of all integrity experiments were also accompanied by uptake studies (Section 3.2). As described before, it was an interesting fact that nearly all of the detectable substance consists of the 8-(*S*) isomeric form, independant whether ET or EC or ergocristinine was used and independant from application compartment. With the assumption that all of the missing substance could be found in the cell lysate over 24 h the alkaloid content accumulates with 12% for ET, 28% for EC and up to 60% for ergocristinine. This high accumulation of the substance could be a possible explanation for the weakened barrier, resulting in the visualized holes in the cell monolayer. Earlier work has determined an accumulation of ergot alkaloids in primary cells [30]. Additionally uptake curves in vivo also show an initial plasma level of ergot alkaloids detectable after a few minutes reaching its maximum after only 1 h and an accumulation of ergot alkaloids is suggested although a low oral bioavailability was shown [18,39].

It has to be highlighted that TEER values do not regenerate during our experiments, like for other substances in the literature [27]. The cells seemed unable to repair the damage induced by the substances. Due to the fact that also ergocristinine shows such a great impact on barrier integrity it underlines the fact that a consideration of toxic effects concerning ergot alkaloids has to be done by always taking both forms into account. The term of biologically inactive compounds in terms of receptor activation does not seem to correlate with secondary effects of ergot alkaloids, which has to be concluded in future research.

### 4.2 Transport properties

Until now data in the literature provided a controversial discussion concerning the permeability of ergot alkaloids, especially ergotamine, across the blood–brain barrier. While one study has shown, that ergotamine is able to reach the CSF [19] another contradicted this [17]. Although this alkaloid has been used in migraine therapy over many years, the transport mechanism remains unclear. Only empirical data are present that ergot alkaloids are able to cross the blood–brain barrier [3]. To evaluate the ability of ergot alkaloids of penetrating the blood–brain barrier, a well-established in vitro model system, consisting of PBCECs was used to determine the transport properties [24].

Based on barrier integrity results, only concentrations of the model substances, retaining the barrier properties were chosen (ET and EM 10 μM and EC and ergocristinine 1 mM). Due to the rearrangement of the substances concerning the 8-(*R*) and 8-(*S*) isomers (exception ergocristinine) the concentrations were calculated as sum parameters. It is not possible to conclude whether one form is transported/ permeated or simply rearranged from the 8-(*R*)/8-(S) equilibrium when reaching the other compartment. Nevertheless, both forms of every ergot alkaloid occur in food and feed. Also, the rearrangement of both forms could possibly take place in vivo and the use of a sum parameter seems to be a very effective way to reflect this situation. All of the tested 8-(*S*) isomeric forms have shown this rearrangement of their equilibrium with the exception of ergocristinine. This is an interesting point since all 8-(*S*) isomers are described to be biologically only weakly active in terms of receptor interaction [11]. Our findings suggest an effect of this isomer on the barrier integrity (Section 4.1). Additionally, the results obtained for ergocristinine (Section 3.2) clearly have shown that the main permeated form, using EC mixture is the 8-(*R*) isomer and the resulting 8-(*S*) isomer in basolateral compartment is a rearrangement from the previous form. This fact could only be shown for EC, due to a stable 8-(*S*) isomeric form. But this also gives the hint for an analogue mechanism for ET and EM. Nevertheless, the mixtures of ET, EM and EC were analyzed as a sum parameter for a suitable in vivo comparison.

As shown in Section 3.2, the isomeric substance mixtures ET, EM and EC were able to cross the formed endothelial cell barrier. The calculated *P*_c_ value for the lipophilic peptide ergot alkaloids ET (a → b: 1.19 ± 0.06 × 10^−5^cm/s) and EC (ab: 2.0570.34 × 10^−5^cm/s) was in a high range (table 1), comparable with the permeation of the amino acid L-leucine in the same model system [40]. EM as the lysergic acid amide showed a comparatively low permeability with *P*_c_ 1.0570.17 × 10^−6^cm/s in a range of the CNS drug morphine [40]. In contrast to this the measured permeability for ^14^C-sucrose was about *P*_c_∼4 × 10^−7^cm/s, a barrier integrity which resembles in vivo observations [25].

The alkaloid morphine shows a comparable *P*_c_ value as EM, while ET and EC permeated much better. The results clearly demonstrate the evidence that all tested compounds are able to cross the blood–brain barrier, while peptide ergot alkaloids are transported in a much higher concentration range. Additionally, at the endpoint of every permeability measurement, the concentrations of substance in cell lysate were measured. Uptake properties correlate with permeability rates of the substance mixtures, with EM showing no measurable cellular uptake, ET mixture (respectively nearly only 8-(*S*) isomeric form in cell lysate) an uptake about 12% and EC (8-(*S*) isomer) an uptake about 28% (Section 4.1). This correlation fits very well to the permeability of the substances, with the exception of ergocristinine, which has shown high uptake properties, but seems unable to leave the cells. Data concerning transport of ergot alkaloids were reported for another peptide ergot alkaloid, ergovaline/ ergovalinine. The transport was elucidated using the Caco-2 model [41]. The permeation of the substance mixture was similar to EC mixture, with 40% of the initial concentration reaching the other compartment after 6 h. This indicates a similar transport behavior for peptide ergot alkaloids and underlines our findings for the blood–brain barrier.

To identify transport properties, a vice versa direction was also determined (b → a). Comparing the permeability coefficients a high significant difference was detectable for EM and a small difference for ET (Fig. 5B). Comparing such coefficients gives the first hint of an active transport to one compartment [42]. In our experiments an evidence for an active transport was given for EM to apical compartment and no differences were detectable for EC. For ET only slight differences with less than factor of 2 were detectable.

Additional experiments with equimolar concentrations of the substances in every compartment should give another hint for an active transport (Fig. 6). As shown in Section 3.3.1, only incubation with EM has shown a significant enrichment in the apical compartment that could not be determined for ET. Incubation with ET and EC only results in a slight decrease in the content for ET and for EC only a basolateral fade of substance (recovery 60%). The missing substance could be detected in cell lysate, accumulating in the cells.

The evidence of an active transport was proven for EM by the use of two different inhibitors against ABC transporters. EM and ET are known in the literature to be Pgp inhibitors, with the peptide ergot alkaloids being more potent [43]. To investigate influence on EM-transport, two different inhibitors PSC and FTC were used. While PSC inhibits Pgp [35], FTC is an inhibitor for the breast cancer-resistant protein (BCRP/ABCG2) [36]. The results show that with the use of PSC only a small setback of transport properties could be measured. With the use of FTC the active transport process was totally inhibited as shown in Section 3.3.2. This indicates that EM is a substrate for BCRP/ABCG2 transporter, while peptide ergot alkaloids do not seem to be influenced (data not shown). The results obtained with PSC could be explained due to a competitive reaction of both substances/ inhibitors PSC and EM, since EM is also an inhibitor for Pgp. A possible side effect of ergot alkaloids acting as the inhibitors for efflux proteins and therefore influencing the transport of other xenobiotics could also not be excluded but was not tested in this study.

The identified active transport to the apical compartment of EM is also reflected by the different *P*_c_ values, as EM shows a significantly lower value compared with peptide ergot alkaloids ET and EC. These different permeation properties seem to be easily explained due to the structure of the molecules. While peptide ergot alkaloids are highly lipophilic compounds, lysergic acid amides are slightly better soluble. Due to the polarity of the molecules a diffusion of ET and EC could be possible. Both isomeric mixtures permeated well to both compartments, depending on the application side. But the results obtained with ergocristinine indicate no transcellular transport, since no substance could be detected after application in the other compartment (a → or → a ). Instead, this gives a hint for a stereoselective transporter which is only able to transport 8-(*R*) isomers, like reported for amino acids [44]. Nevertheless, even if no 8-(*S*) isomer is transported, the substance could be rearranged in the other compartment, resulting in a ''permeation'' of the 8-(*S*) isomer. As shown in Section 4.1 both forms also seemed to have an effect on barrier integrity, consequently the 8-(*S*) isomers also have a very high relevance for ergot alkaloid research.

Overall, our data confirm the findings of Ala-Hurula et al. [19]. Peptide ergot alkaloids like EC and ET are able to pass the blood–brain barrier reaching the CNS in a high concentration. Both substances are in a sum referred as 58% of the sclerotia alkaloid content [37,38], which makes them important for a risk assessment. Due to the receptor interaction of ergot alkaloids, requiring only concentration in a nanomolar range, even small concentrations could have a possible influence. These concentrations could even be below detection limit as suggested in the literature [20]. The contradictory findings of Hovdal et al. [17] to those of Ala Hurula [19] were assumed in the literature as a problem in the detection method of the ergot alkaloids [20] and could consequently not be confirmed with our experiments.

Our results using PBCECs as an in vitro model system clearly indicates a penetration of ergot alkaloids through the blood–brain barrier. Although an active transport could be identified for EM, the ability to reach the brain is still comparable to CNS active substances. In contrast to these results, no active transport properties could be identified for ET and EC. Within a few hours the substances were able to cross the blood–brain barrier, having impact on the brain; inducing toxic effects, interact with receptor systems or accumulate there. Furthermore, the results have shown evidence that only 8-(*R*) isomers are able to permeate with the example of ergocristinine as 8-(*S*) isomer being unable to cross the barrier. However, a high accumulation in cells was detected and an influence on barrier integrity was identified. All the results indicate a complex mode of action of ergot alkaloids, even from the 8-(*S*) isomers, which is still not described. Small concentrations, like those found in food and feed could inflict a wide range of effects, due to very effective transport/permeation properties and are therefore important for consideration of toxic effects induced by ergot alkaloids.
